# A Practical Treatise on Diseases of the Skin

**Published:** 1866-12

**Authors:** 


A Practical Treatise on Diseases of the Skin. By J. Moore Neligan, M.D., M.R.I.A. etc. Fifth American, from the second revised and enlarged Dublin edition. By T. W. Belcher, M.A., M.D., Dub.; B.M., M.A., Oxon., etc., etc., etc., etc. Philadelphia: Henry C. Lea. 1866.

This is a duodecimo volume of 462 pages. The editor, Dr. Belcher, has added to this edition several pages of new and valuable matter, a more copious index, the derivation and meaning of technical terms, and the consideration of several diseases not referred to in former editions. As a brief treatise on diseases of the skin, it is one of the best now in the hands of the profession, and one of the most convenient, either for reference or study.

For sale by S. C. GRIGGS & Co., Lake Street.

